# Impact of Live *Ligilactobacillus salivarius* CCFM1332 and Its Postbiotics on *Porphyromonas gingivalis* Colonization, Alveolar Bone Resorption and Inflammation in a Rat Model of Periodontitis

**DOI:** 10.3390/microorganisms13071701

**Published:** 2025-07-20

**Authors:** Qing Hong, Yu Ren, Xin Tang, Bingyong Mao, Qiuxiang Zhang, Jianxin Zhao, Shumao Cui, Zhenmin Liu

**Affiliations:** 1 State Key Laboratory of Dairy Biotechnology, Shanghai Engineering Research Center of Dairy Biotechnology, Dairy Research Institute, Bright Dairy & Food Co., Ltd., Shanghai 200436, China; hongqing1@brightdairy.com; 2 State Key Laboratory of Food Science and resources, Jiangnan University, Wuxi 214122, China; yu.ren@teshiyuan.com (Y.R.); xintang@jiangnan.edu.cn (X.T.); maobingyong@jiangnan.edu.cn (B.M.); zhangqx@jiangnan.edu.cn (Q.Z.); zhaojianxin@jiangnan.edu.cn (J.Z.); 3 School of Food Science and Technology, Jiangnan University, Wuxi 214122, China

**Keywords:** periodontitis, *Ligilactobacillus salivarius*, postbiotics, inflammation, alveolar bone resorption

## Abstract

Periodontitis is a chronic inflammatory disease caused by periodontopathic bacteria such as *Porphyromonas gingivalis* (*P. gingivalis*), which leads to alveolar bone destruction and systemic inflammation. Emerging evidence suggests that probiotics may mitigate periodontal pathology. To systematically evaluate the alleviative effects and mechanisms of different forms of probiotics, including live bacteria and postbiotics, on periodontitis, we first screened and identified *Ligilactobacillus salivarius* CCFM1332 (*L. salivarius* CCFM1332) through in vitro antibacterial and anti-biofilm activity assays. Subsequently, we investigated its therapeutic potential in a rat model of experimental periodontitis. The results demonstrated that both live *L. salivarius* CCFM1332 (PL) and its postbiotics (PP) significantly reduced the gingival index (GI) and probing depth (PD) in rats, while suppressing oral colonization of *P. gingivalis*. Serum pro-inflammatory cytokine levels were differentially modulated: the PL group exhibited reductions in interleukin-17A (IL-17A), interleukin-6 (IL-6), and interleukin-1β (IL-1β) by 39.31% (*p* < 0.01), 17.26% (*p* < 0.05), and 14.74% (*p* < 0.05), respectively, whereas the PP group showed decreases of 34.79% (*p* < 0.05), 29.85% (*p* < 0.01), and 19.74% (*p* < 0.05). Micro-computed tomography (Micro-CT) analysis demonstrated that compared to the periodontitis model group (PM), the PL group significantly reduced alveolar bone loss (ABL) by 30.1% (*p* < 0.05) and increased bone volume fraction (BV/TV) by 49.5% (*p* < 0.01). In contrast, while the PP group similarly decreased ABL by 32.7% (*p* < 0.05), it resulted in a 40.4% improvement in BV/TV (*p* > 0.05). Histological assessments via hematoxylin and eosin (H&E) and tartrate-resistant acid phosphatase (TRAP) staining confirmed that both the PL group and the PP group alleviated structural damage to alveolar bone-supporting tissues and reduced osteoclast-positive cell counts. This study suggests that live *L. salivarius* CCFM1332 and its postbiotics reduce alveolar bone resorption and attachment loss in rats through antibacterial and anti-inflammatory pathways, thereby alleviating periodontal inflammation in rats.

## 1. Introduction

Periodontitis, ranked as the sixth most prevalent disease worldwide, is a chronic inflammatory disease characterized by progressive destruction of periodontal tissues, including gingival bleeding, periodontal pocket formation, connective tissue attachment loss, and alveolar bone resorption [1,2]. According to the Global Burden of Disease Study (GBD 2019), approximately 1.1 billion individuals worldwide suffer from severe periodontitis, with an adult prevalence rate of 10.6% [3]. Left untreated, this condition may lead to tooth loss, masticatory dysfunction, and malnutrition, severely compromising quality of life and imposing substantial public health and economic burdens [4,5]. Furthermore, severe periodontitis is bidirectionally linked to systemic diseases such as diabetes and cardiovascular disorders, exacerbating both oral and systemic health challenges [6,7].

The pathogenesis of periodontitis is driven by subgingival microbial dysbiosis, where keystone pathogens such as *Porphyromonas gingivalis* subvert host immunity via lipopolysaccharide (LPS)-mediated activation of the complement 5a receptor 1-Toll-like receptor 2 (C5aR1-TLR2) crosstalk pathway [8]. This triggers the phosphatidylinositol 3-Kinase/protein kinase B/mammalian target of rapamycin (PI3K/Akt/mTOR) cascade that disrupts innate immune barriers and promotes the release of pro-inflammatory cytokines (e.g., interleukin-1β (IL-1β) and tumor necrosis factor alpha (TNF-α)) [9,10,11]. Subsequent activation of matrix metalloproteinases and receptor activator of nuclear factor kappa-B ligand/osteoprotegerin (RANKL/OPG)-mediated osteoclast overactivation culminates in collagen degradation and alveolar bone resorption [12].

Current clinical treatment primarily relies on subgingival scaling and root planing (SRP), which fails to address microbial dysbiosis or prevent disease recurrence [13,14,15]. Recent advances in microbiome research have highlighted probiotics as a promising therapeutic strategy for periodontal diseases by leveraging their ecological modulation capabilities [16,17]. Probiotics exert their effects through multiple mechanisms: they form biological antagonism by competing with pathogens for nutritional substrates and adhesion sites [18]; secrete antimicrobial substances such as bacteriocins and hydrogen peroxide to directly inhibit the proliferation of pathogens [19]; regulate the balance of epithelial cell proliferation and apoptosis to repair barrier function [20]; and activate dendritic cells and the differentiation of regulatory T cells to reshape mucosal immune responses [16]. This process suppresses pro-inflammatory cytokines (e.g., IL-1β, TNF-α) and attenuates inflammatory cascades [21].

Notably, specific probiotic strains have demonstrated significant therapeutic efficacy in clinical studies. For example, *Limosilactobacillus reuteri* has been proven to attenuate the virulence of key periodontal pathogens, modulate the immune-inflammatory process, and promote periodontal tissue repair [22]. Other probiotic species have also been observed to improve periodontal treatment outcomes, including *Ligilactobacillus salivarius* [23], *Lactobacillus fermentum* [24], and *Lacticaseibacillus rhamnosus* [25]. However, live probiotics face limitations including poor storage stability, low oral colonization efficiency, and the risk of ectopic infection. In contrast, postbiotics, as inactivated microbial metabolites, offer advantages such as high chemical stability, colonization independence, and multitarget activity, showing promise in anti-inflammatory and immunomodulatory applications [26,27]. However, the comparative efficacy of postbiotics versus live probiotics in periodontitis remains unclear, and their therapeutic mechanisms require further exploration [28,29].

In this study, we aimed to screen probiotic strains with anti-*P. gingivalis* activity in vitro and evaluate the therapeutic effects of both live cells and postbiotics derived from the screened strain on periodontitis using a rat model. Our findings provide insights into the potential of postbiotics as alternatives to traditional probiotics for periodontal therapy.

## 2. Materials and Methods

### 2.1. Strain Preparation

*P. gingivalis* strain ATCCW83 was purchased from the Guangdong Microbiological Culture Collection Centre (GDMCC, Guangzhou, China). *P. gingivalis* was cultured anaerobically at 37 °C for 48 h in brain heart infusion (BHI) medium (Hi-Tech, Qingdao, China) supplemented with 0.05% hemin and 0.1% vitamin K1 (Solarbio, Beijing, China) (BHI-H). After two consecutive subcultures, the bacterial suspension was centrifuged at 3000× *g* for 10 min at 4 °C and adjusted to 1 × 10^5^ CFU/mL for in vitro experiments or 1 × 10^9^ CFU/mL for in vivo studies.

Five strains of *L. salivarius* (FHNXY73M9, FGSYC47M10, CCFM1332, FXJWS6M4, and FJSWX10-2) and four strains of *L. plantarum* (QS6-12, CCFM242, DL2-1, and CCFM10) were obtained from the Culture Collection of Food Microorganisms of Jiangnan University. These strains were isolated from fermented foods, fecal or oral samples of healthy individuals, or animal feces. The taxonomic identity was confirmed by 16S rRNA sequencing, genome sequencing, and biochemical profiling (strain characterization data are available from the corresponding author upon request; these strains will be provided to qualified researchers for non-commercial academic research purposes under reasonable conditions). The strains were cultured in MRS broth at 37 °C under aerobic conditions for 20 h. For in vitro studies, the cultured bacteria (1 × 10^9^ CFU/mL) were centrifuged at 8000× *g* for 15 min at 4 °C, and the supernatant was sterile-filtered (0.22 μm) to obtain cell-free supernatant (CFS) [30]. For in vivo experiments, the same number of bacteria were collected after centrifugation and resuspended in PBS to 1 × 10^9^ CFU/mL [31]. The same number of bacteria were lysed via high-pressure homogenization at 1200 bar for 10 min to prepare postbiotics.

### 2.2. Assessment of Antibacterial Activity

The antibacterial activity of *Lactobacillus* CFS was evaluated using the agar diffusion method with an Oxford cup [30]. *P. gingivalis* was cultured on BHI-H agar plates supplemented with 5% sterile defibrinated sheep blood (BHI-H-B). Four sterile Oxford cups were placed on each plate. Into each cup, 150 μL of CFS was added. Controls included 0.02% chlorhexidine (CHX) as the positive control and MRS broth as the negative control. Plates were incubated anaerobically at 37 °C for 48 h, and the diameter of inhibition zones (DIZ) was measured to quantify antibacterial activity.

### 2.3. Assessment of Anti-Biofilm Activity

The ability of *Lactobacillus* CFS to inhibit *P. gingivalis* biofilm formation was assessed using a crystal violet staining assay in 96-well polystyrene microtiter plates [32]. Overnight cultures of *P. gingivalis* in BHI-H broth were adjusted to 1 × 10^5^ CFU/mL and transferred to 96-well plates. After 24 h of anaerobic incubation at 37 °C, 80 μL of CFS was added to each well and incubated for an additional 24 h. MRS broth served as the negative control, while 0.02% CHX was used as the positive control. Planktonic cells were removed by gentle washing with PBS, and adherent biofilms were fixed with 200 μL of 0.1% crystal violet for 30 min. After two PBS washes, bound dye was solubilized with 100 μL of 95% ethanol, and absorbance at 600 nm (OD_600_) was measured using a microplate reader (Thermo Fisher Scientific, Waltham, USA). The percentage inhibition of biofilm formation was calculated as: Inhibition (%) = (OD_NC_ − OD_sample_)/OD_NC_ × 100%.

### 2.4. Animal Experimental Design

Five-week-old male Wistar rats (purchased from Beijing Vital River Laboratory Animal Technology Co., Ltd., Beijing, China) were housed under a 12 h light/dark cycle at 22–24 °C with free access to food and water. All experimental procedures were approved by the Institutional Animal Care and Use Committee (IACUC) of the Jiangsu Institute of Parasitic Diseases (IACUC-JIPD-2024-105).

After a 7-day acclimation period, 30 rats were randomly divided into five groups (*n* = 6 per group): no-treatment control group (NT), periodontitis model group (PM), periodontitis + CHX group (PC), periodontitis + live *L. salivarius* CCFM1332 group (PL), and periodontitis + postbiotics of *L. salivarius* CCFM1332 group (PP).

The periodontitis model was established according to Ye et al. [31]: On day 1, rats were anesthetized with 100 mg/kg ketamine. A 0.22 mm orthodontic wire was ligated around the left maxillary second molar, ensuring placement into the gingival sulcus. Postoperatively, rats received ampicillin-supplemented water (125 μg/mL) for 3 days to suppress endogenous flora. From days 8–21, the ligated site was rinsed with *P. gingivalis* (1 × 10^9^ CFU/mL) using a syringe five times per week. After each inoculation, rats were fasted for 30 min to enhance bacterial colonization.

From days 22–35, the PM, PC, PL, and PP groups received topical applications of 0.5 mL PBS, 0.02% CHX, live *L. salivarius* CCFM1332, or postbiotics of *L. salivarius* CCFM1332, respectively, six times per week. Rats were fasted for 30 min post-treatment.

### 2.5. Determination of P. gingivalis Colonization

On days 21, 28, and 35 of the experiment, saliva samples were collected from the ligated area using sterile cotton swabs applied with uniform pressure for 10 s and immediately transferred to 2 mL sterile centrifuge tubes. Within 4 h, 1 mL of sterile saline was added to each tube, followed by vortex mixing. Serial dilutions from 10^−1^ to 10^−6^ were then prepared. Then, 1 mL of each dilution was inoculated onto BHI-H-B agar using the pour plate method and incubated anaerobically at 37 °C for 5–7 days. Colony counts were expressed as Log CFU/mL [33]. *P. gingivalis* colonies were identified by their characteristic black pigmentation and confirmed using PCR with species-specific primers.

### 2.6. Determination of Gingival Index (GI) and Probing Depth (PD)

At the end of the experiment, rats were anesthetized for assessment of the gingival index (GI) and probing depth (PD) on the ligated-side second molar. GI was scored (0–3) following Löe H’s criteria [34], where 0 = healthy gingiva, 1 = mild inflammation without bleeding, 2 = moderate inflammation with bleeding, and 3 = severe inflammation with ulceration. PD was measured as the distance from the gingival margin to the pocket base using a periodontal probe, with three palatal measurement sites (mesio-, mid-, and distopalatal) averaged for final values [35].

### 2.7. Measurement of Inflammatory Cytokines

At the end of the experiment, rats were anesthetized, and blood samples were collected from the abdominal aorta. The blood was allowed to clot for 1 h at room temperature, followed by centrifugation at 5000 rpm for 10 min at 4 °C to isolate the serum. Serum levels of TNF-α, IL-1β, interleukin-6 (IL-6), interleukin-8 (IL-8), interleukin-17A (IL-17A), and interleukin-10 (IL-10) were quantified using an enzyme-linked immunosorbent assay (ELISA) kit (SenBeiJia Biological Technology, Nanjing, China) according to the manufacturer’s instructions.

### 2.8. Micro-Computed Tomography Imaging

Following euthanasia, the maxillas from the ligation side were separated and fixed in 4% paraformaldehyde for 48 h, rinsed with PBS buffer solution, and air-dried [36]. The prepared rat maxillas underwent micro-computed tomography (Micro-CT) (Quantum GX2; PerkinElmer, Hopkinton, MA, USA) scanning with the following parameters: 90 kV, 88 µA, a pixel size of 18 µm, and a 180° imaging rotation [33]. The regions of interest were standardized at the bifurcation of the second molars using CTAn software (Skyscan v.1.17.7.2) to calculate the bone volume/total volume (BV/TV). Alveolar bone loss (ABL) was quantified as the distance between the cemento-enamel junction (CEJ) and the alveolar crest (ABC). ABL was measured at six sites around the second molar: distal, mesial, and median of the buccal and lingual [37].

### 2.9. Histopathological Analysis

After Micro-CT scanning, the maxillae were decalcified in ethylenediaminetetraacetic acid (EDTA) solution (pH 7.8) for 10 days. The tissues were embedded in paraffin using an embedding machine for solidification. Following embedding, 4 μm thick sagittal sections were cut along the long axis of the molars and stained with hematoxylin and eosin (H&E). The sections were then examined under high magnification with a digital pathology slide scanner.

### 2.10. TRAP Staining

From the same paraffin block, 4 µm thick sections were prepared for tartrate-resistant acid phosphatase (TRAP) staining. TRAP-positive multinucleated cells exhibiting a wine-red coloration adjacent to the alveolar bone surface were identified as active osteoclasts using a slide scanner at high magnification. For quantification, five randomly selected fields of view per section were analyzed at 15× magnification, and osteoclast numbers were counted [38].

### 2.11. Statistical Analysis

Data were statistically analyzed using SPSS 25.0. The data were tested for normality by the Shapiro–Wilk test. Parametric data were analyzed using an independent *t* test. Nonparametric data were analyzed using the Mann–Whitney test. In all data analyses, *p* < 0.05 indicates statistically significant. GraphPad Prism 8.0 software was utilized for graphing. Results are presented as the mean ± standard error of the mean (Mean ± SEM).

## 3. Results

### 3.1. Antibacterial Activity of Lactobacillus Against P. gingivalis

The inhibitory effects of various *Lactobacillus* strains on *P. gingivalis* were evaluated using a co-culture assay. As shown in Table 1, one strain of *L. salivarius* and four strains of *L. plantarum* exhibited significant growth inhibition of *P. gingivalis*. Among these, *L. salivarius* CCFM1332 demonstrated the strongest antibacterial activity, with a DIZ of 20.15 mm.

### 3.2. Anti-Biofilm Activity of Lactobacillus to P. gingivalis

Bacterial biofilms are complex, three-dimensional structures formed by aggregated microbial communities. These matrices protect embedded bacterial cells from environmental stressors and enhance resistance to antibiotics or host immune responses [39].

To evaluate the anti-biofilm potential of *Lactobacillus* strains against *P. gingivalis*, their ability to disrupt preformed biofilms was quantified. As shown in Table 2, all nine *Lactobacillus* strains exhibited an inhibition rate of over 80%. Notably, four *L. salivarius* strains (FZJTZ1M1, FHNXY73M9, CCFM1332, and FJSWX10-2) exhibited inhibition rates exceeding 90%, with *L. salivarius* CCFM1332 showing the highest inhibition rate (92.98%). Collectively, these findings indicate that *L. salivarius* CCFM1332 exhibits the strongest efficacy in suppressing both planktonic growth and biofilm formation of *P. gingivalis*. Given its potent inhibitory effects against periodontal pathogens, this strain was selected as the target for in vivo experiments to further investigate its anti-periodontitis effects.

### 3.3. The Effect of L. salivarius CCFM1332 on the Body Weight of Rats

To determine whether periodontitis affected rat body weight, weekly measurements were recorded (Table 3). During the study period, all groups exhibited a steady increase in body weight. The NT group (no-treatment control) exhibited marginally higher weight gain than the PM group (periodontitis model) and intervention groups, including PC (periodontitis treated with CHX), PL (periodontitis treated with live *L. salivarius* CCFM1332), and PP (periodontitis treated with postbiotics of *L. salivarius* CCFM1332) groups. However, no statistically significant differences in body weight were observed among any groups during the experimental period.

### 3.4. The Effect of L. salivarius CCFM1332 on the GI and PD of Rats

To evaluate the efficacy of live *L. salivarius* and its postbiotics in mitigating periodontal inflammation in rats, GI and PD were assessed. As presented in Table 4, the PM group exhibited significantly elevated PD and GI compared to the NT group (*p* < 0.01). The PD was notably decreased in the PC and PL groups compared to the PM group, with reductions to 0.58 (*p* < 0.01) and 0.85 (*p* < 0.05), respectively. Furthermore, the GI was significantly lower in the PC (0.50), PL (0.50), and PP (0.60) groups in comparison to the PM group (1.50).

### 3.5. The Effect of L. salivarius CCFM1332 on Serum Inflammatory Cytokines of Rats

**Figure 1:**
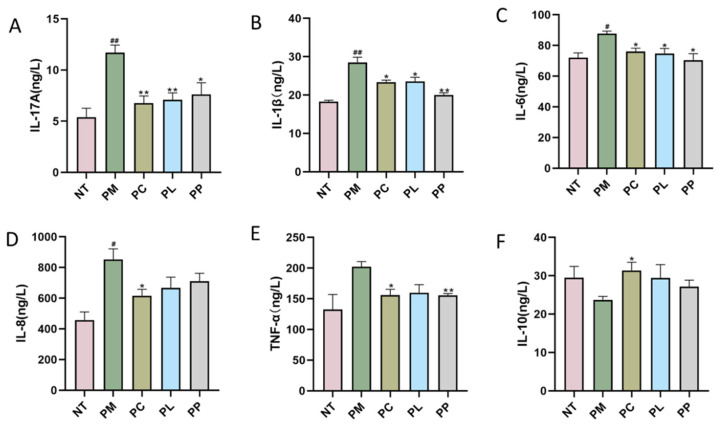
Content of serum IL-17A (**A**), IL-1β (**B**), IL-6 (**C**), IL-8 (**D**), TNF-α (**E**), IL-10 (**F**) in rats. ^#^ *p* < 0.05 vs. NT group; ^##^ *p* < 0.01 vs. NT group; * *p* < 0.05 vs. PM group; ** *p* < 0.01 vs. PM group.

Quantitative analysis of serum inflammatory cytokines (IL-1β, IL-6, IL-8, IL-17A, TNF-α) and the anti-inflammatory cytokine IL-10 revealed distinct patterns across groups (Figure 1). Compared to the NT group, the PM group exhibited significant increases in pro-inflammatory cytokines: IL-17A (+117%, *p* < 0.01), IL-1β (+56%, *p* < 0.01), IL-6 (+22%, *p* < 0.05), and IL-8 (+86%, *p* < 0.05), indicating systemic inflammatory activation. All intervention groups significantly reduced IL-17A, IL-1β, and IL-6 levels compared to the PM group. Specifically, PC group reduced IL-17A by 42.13% (*p* < 0.01), IL-1β by 17.99% (*p* < 0.05), and IL-6 by 13.27% (*p* < 0.05); PL group decreased these cytokines by 39.31% (*p* < 0.01), 17.26% (*p* < 0.05), and 14.74% (*p* < 0.05), respectively; and PP group achieved reductions of 34.79% (*p* < 0.05), 29.85% (*p* < 0.01), and 19.74% (*p* < 0.05). Additionally, both PC (*p* < 0.05) and PP (*p* < 0.01) groups further suppressed TNF-α levels. Notably, the PC group uniquely reduced IL-8 by 27.74% (*p* < 0.05) and elevated IL-10 by 32.25% (*p* < 0.05), effects absent in PL or PP groups.

### 3.6. The Effect of L. salivarius CCFM1332 on the Load of P. gingivalis in the Oral Cavity of Rats

**Figure 2:**
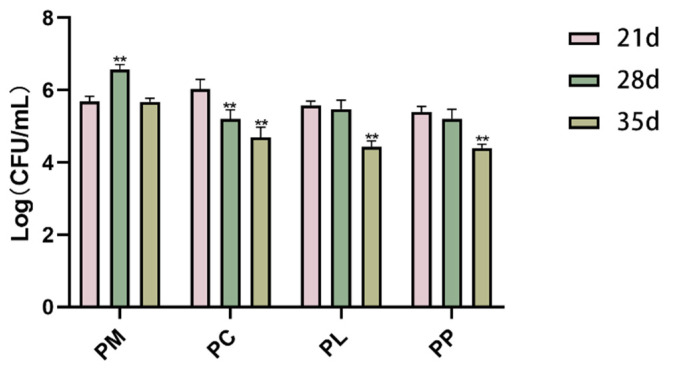
Changes in oral *P. gingivalis* load in rats. ** *p* < 0.01 vs. 21d.

After a two-week (21 d) rinse with *P. gingivalis*, the rats exhibited successful bacterial colonization with a *P. gingivalis* count exceeding 5.0 Log (CFU/mL) in their oral cavities (Figure 2). Following one week (28 d) of intervention, the PC group showed a significant reduction in *P. gingivalis* count (*p* < 0.01), with a further pronounced decrease observed after two weeks of intervention (35 d). Similarly, after two weeks (35 d) of intervention, the counts of *P. gingivalis* in the PL (*p* < 0.01) and PP (*p* < 0.01) groups were significantly reduced, from 5.58 to 4.44 and 5.40 to 4.40 Log (CFU/mL), respectively. In conclusion, after the two-week intervention period, all groups showed a reduction in *P. gingivalis* count by at least one order of magnitude.

### 3.7. The Effect of L. salivarius CCFM1332 on Alveolar Bone Resorption of Rats

**Figure 3:**
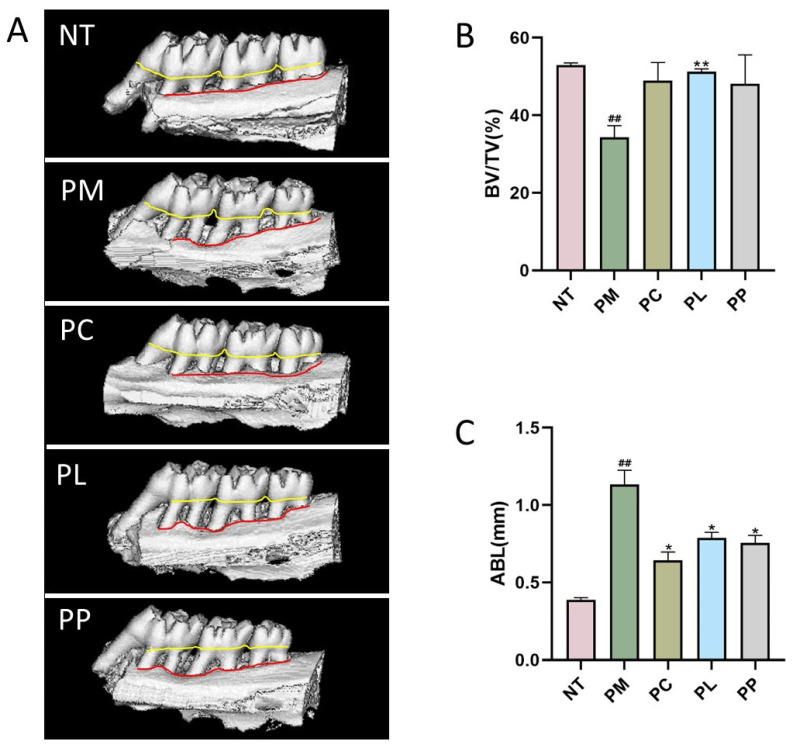
Changes in the microstructure of the maxilla. (**A**) Micro-CT image of the maxilla, the yellow line shows the cemento-enamel junction, the red line shows the alveolar bone crest; (**B**) comparison of the bone-to-tissue volume (BV/TV) ratio around the distal root of the mandibular first molar; (**C**) the average distance from the palatal and mesiobuccal CEJ to the ABC. ^##^ *p* < 0.01 vs. NT group; * *p* < 0.05 vs. PM group; ** *p* < 0.01 vs. PM group.

Micro-CT enables three-dimensional imaging of the maxilla without causing damage to the alveolar bone, providing an assessment of alveolar bone loss due to periodontitis. In this experiment, the PM group displayed marked alveolar bone loss, characterized by enlarged interdental spaces and evident exposure of the molar furcations, confirming the successful establishment of the periodontitis model. Following intervention in each group, there was a noticeable reduction in alveolar bone height and decreased exposure of the apical furcation, as depicted in Figure 3A.

Quantitative analysis of bone microstructure revealed significant differences between groups. The bone volume fraction (BV/TV) in the PM group (34.30%) was markedly lower than that in the no-treatment (NT) control group (52.96%, *p* < 0.01). Notably, the PL group demonstrated substantial bone regeneration with a BV/TV ratio of 51.26% (*p* < 0.01 vs. PM), approaching the level of healthy controls. While the PC (48.99%) and PP (48.15%) groups showed a tendency toward increased BV/TV, these changes did not reach statistical significance (Figure 3B).

ABL, representing the height of bone resorption, was measured from the three-dimensional reconstructions of alveolar bone (Figure 3C). The PM group displayed significantly greater ABL (1.13 mm) compared to the NT group (0.39 mm, *p* < 0.05). This indicates that the periodontitis model induced significant alveolar bone loss. Therapeutic interventions markedly attenuated bone resorption, with ABL reductions reaching 43.36% (0.64 mm) in the PC group (*p* < 0.05), 30.09% (0.79 mm) in the PL group (*p* < 0.05), and 32.74% (0.76 mm) in the PP group (*p* < 0.05), all demonstrating statistically significant differences versus the PM group (all *p* < 0.05) (Figure 3C).

### 3.8. The Effect of L. salivarius CCFM1332 on the Morphological Structure of Alveolar Bone Tissue of Rats

**Figure 4:**
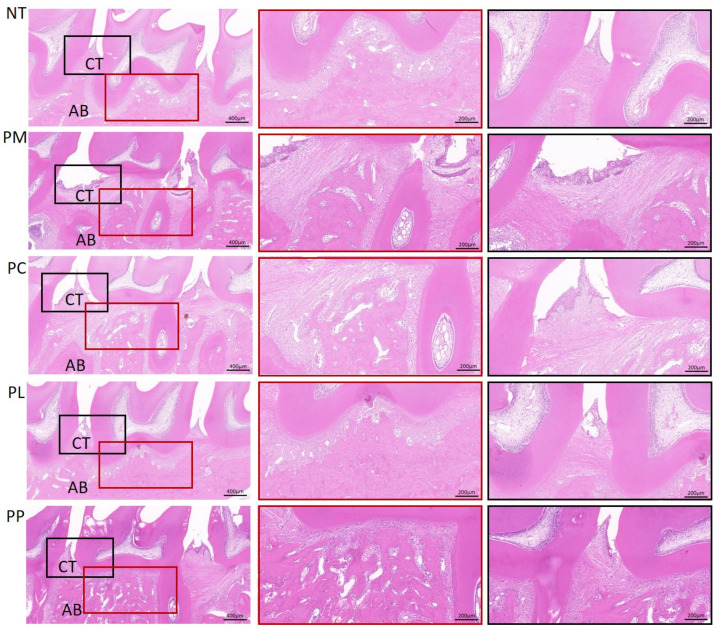
Histology of alveolar bone disease in rats. AB: alveolar bone, CT: connective tissue; the red box selects the furcation area of the second molar, and the black box selects the connective tissue between the first and second molars (scale bars, 400 μm, 200 μm, 200 μm).

H&E staining was conducted on the maxillary alveolar bone from the ligation side of rats to evaluate the extent of pathological damage (Figure 4). The NT group demonstrated normal periodontal tissue and alveolar bone architecture, characterized by a tight junctional epithelial attachment to the tooth surface, absence of attachment loss, and an intact, orderly arrangement of periodontal ligament fibers. Conversely, the PM group exhibited significant downward growth of the junctional epithelium, dissolution of periodontal fibers, pronounced attachment loss, and alveolar bone resorption. In contrast, the PC, PL, and PP groups displayed periodontal pathological conditions closer to those of the control group, with relatively intact gingival epithelial structures and a less severe degree of attachment loss.

### 3.9. The Effect of L. salivarius CCFM1332 on Osteoclasts in the Alveolar Bone of Rats

**Figure 5:**
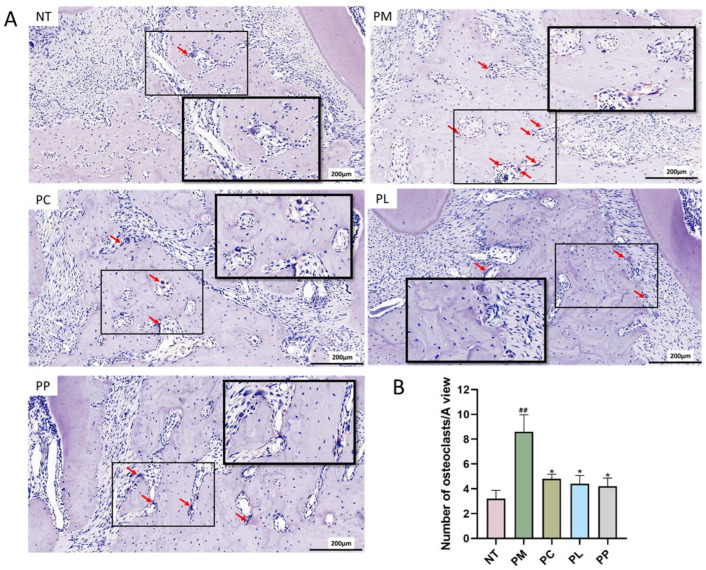
Changes in the number of osteoclasts in the alveolar bone of rats. (**A**): TRAP staining (scale bar, 200μm); (**B**): The number of osteoclasts. The red arrows point to TRAP-positive multinucleated cells; the black box indicates the region shown at higher magnification. ^##^ *p* < 0.01 vs. NT group; * *p* < 0.05 vs. PM group.

The effects of various intervention groups on osteoclastogenesis in the alveolar bone of periodontitis-afflicted rats were evaluated using TRAP staining (Figure 5A). The PM group exhibited a higher osteoclast count following periodontitis induction compared to the NT group. Specifically, the osteoclast count in the PM group (8.60 per field of view) was significantly elevated compared to the NT group (3.20 per field of view) (*p* < 0.05). After two weeks of intervention, a significant reduction in osteoclast numbers was observed in the PC (*p* < 0.05), PL (*p* < 0.05), and PP (*p* < 0.05) groups, with counts decreasing to 4.80, 4.40, and 4.20 per field of view, respectively.

## 4. Discussion

The genus *Lactobacillus* has demonstrated therapeutic potential in periodontitis management through multifaceted mechanisms, with pathogen suppression being a key mode of action [31,40]. Indeed, our in vitro screening identified *L. salivarius* CCFM1332 as a potent antagonist against *P. gingivalis*. Compared with the untreated control group, it achieved a DIZ of 20.15 mm against *P. gingivalis* and a biofilm biomass inhibition rate of 92.98%. Furthermore, through animal experiments, we evaluated the therapeutic effects of the active bacteria and postbiotics of this strain on experimental periodontitis. The *periodontitis* models were established according to the method of a previous study, which can better simulate the development process of human periodontitis [31]. Successful pathogen colonization (>5.0 Log (CFU/mL)) induced hallmark clinical features: GI of 1.50, PD increase from 0.24 mm to 1.24 mm, and ABL of 1.13 mm. Wang et al. [41] also observed similar inflammatory progression in a rat periodontitis model established using the same method.

Probiotics, as living microorganisms, can inhibit the growth of periodontal pathogens by competing with them for living space and nutrients [26,42]. Our findings reveal that live *L. salivarius* CCFM1332 administration reduced *P. gingivalis* colonization by 1.14 Log (CFU/mL) (*p* < 0.01) within 14 days, demonstrating faster pathogen suppression than the 30-day regimen required by *L. gasseri* in prior studies [43]. Notably, postbiotics derived from this strain achieved comparable inhibitory effects (1.0 Log reduction), likely mediated through retained antimicrobial metabolites such as bacteriocins and organic acids. These results align with emerging evidence that specific postbiotic components maintain bioactive properties independent of microbial viability [44,45,46].

In our experiments, we observed elevated levels of serum pro-inflammatory cytokines (IL-17A, IL-1β, IL-6, IL-8) associated with periodontitis. This increase is attributed to *P. gingivalis* biofilm formation on tooth and gum surfaces, which, upon continuous accumulation, interacts with complement or pattern recognition receptors (PRRs) to trigger the innate immune response [47]. This interaction leads to an overactivation of immune cells, resulting in the release of inflammatory mediators into the bloodstream and the establishment of a chronic, low-grade inflammatory state [12,48]. Numerous studies have confirmed that individuals with periodontitis exhibit higher serum levels of inflammatory markers such as IL-1β [49,50,51], TNF-α [52,53], IL-17A [54,55], IL-6 [56], and lower levels of the anti-inflammatory cytokine IL-10 [57,58] compared to healthy controls. Therefore, the attenuation of the inflammatory status by *L. salivarius* CCFM1332 might have been related to its inhibition of *P. gingivalis*. Supporting our findings, Nie et al. reported a decrease in IL-6 levels in plasma following supplementation with *L. salivarius* ZK-88 [59]. However, *L. salivarius* CCFM1332 was less effective in regulating IL-8 and IL-10 compared to CHX. IL-8 is a key chemokine primarily secreted by gingival epithelial cells, macrophages, and fibroblasts upon bacterial stimulation. It mediates local inflammatory responses by recruiting and activating neutrophils [60]. IL-10, conversely, induces immunosuppression by altering macrophage function. During inflammation, IL-10 binds to receptors on the macrophage surface and inhibits inflammatory factor secretion [61]. As a broad-spectrum antimicrobial agent, CHX reduces the stimulation of gingival tissues by bacterial metabolites (such as lipopolysaccharide (LPS) and toxins) through the inhibition of pathogenic bacteria growth [62]. This indirectly attenuates the host’s excessive inflammatory response, thereby achieving the regulation of IL-8 and IL-10. In contrast, the regulatory effect of *L. salivarius* CCFM1332 on these cytokines was inferior to that of CHX, which may be attributed to its longer onset time for bacterial inhibition. As shown in Figure 2, CCFM1332 required two weeks of intervention before significantly reducing *P. gingivalis* load, whereas CHX demonstrated a significant inhibitory effect against this bacterium after only one week of intervention.

Periodontal inflammation induced by *P. gingivalis* is characterized by clinical manifestations such as gingival erythema and bleeding, a phenomenon attributed to bacterial virulence factors and dysregulated host immune responses [63]. Concurrently, microbial biofilm accumulation and sustained host–pathogen interactions exacerbate periodontal tissue destruction, leading to progressive deepening of periodontal pockets [64]. In our experimental model, these pathophysiological processes were reflected in significantly elevated PD and GI values, consistent with mild to moderate periodontal inflammation. Notably, both live *L. salivarius* CCFM1332 and its postbiotics demonstrated therapeutic efficacy in attenuating these clinical parameters, aligning with previous findings by Nie et al. and Matsuoka et al. using *L. salivarius* strains in periodontitis management [59,65].

Histopathological analysis further revealed hallmark features of periodontitis in model groups, including connective tissue attachment loss and disorganization of periodontal ligament fibers. These structural alterations may be mechanistically linked to the observed upregulation of IL-1β, a pro-inflammatory cytokine known to amplify tissue degradation through multiple pathways. IL-1β could activate neutrophils and macrophages, thereby inducing the production and release of reactive oxygen species (ROS) and nitric oxide (NO), leading to local tissue damage [66]. Our intervention strategy effectively mitigated these destructive processes, suggesting that *L. salivarius* CCFM1332 and its postbiotics may modulate the IL-1β-mediated inflammatory cascade. The comparable therapeutic outcomes between live probiotics and postbiotics warrant particular attention. This observation implies that specific bioactive metabolites or structural components within the postbiotic fraction may mediate anti-inflammatory effects independent of bacterial viability. Further characterization of these active constituents could advance the development of shelf-stable periodontal therapeutics with enhanced clinical applicability.

The inflammatory cascade in periodontitis drives alveolar bone destruction through complex interactions between cytokine networks and osteoclast activation [67]. Our findings demonstrate that both live *L. salivarius* CCFM1332 and its postbiotic derivatives effectively attenuated periodontitis-induced bone loss and osteoclastogenesis, paralleling the osteoprotective effects reported for other *Lactobacillus* strains [24]. This protective mechanism appears to be mediated through downregulation of key pro-inflammatory mediators, particularly IL-1β, which orchestrates bone resorption via multiple pathways. IL-1β not only enhances MMP-3 production to facilitate leukocyte infiltration and tissue degradation but also stimulates RANKL expression that promotes osteoclast differentiation and activity [68,69]. Notably, the comparable efficacy between live bacteria and postbiotics highlights the potential clinical advantage of using non-viable microbial derivatives. While live probiotics require sustained oral colonization to exert effects, postbiotics may provide immediate bioactive components without colonization challenges. This finding aligns with in vitro evidence demonstrating *L. salivarius*-derived postbiotics inhibit osteoclastogenesis through direct suppression of RANKL signaling pathways [45]. The bone microarchitecture preservation observed in micro-CT analysis (BV/TV restoration from 34.30% to 48.15% in the PP group) further confirms the translational potential of this intervention strategy. In this study, the number of osteoclasts in the alveolar bone of the periodontitis model group had significantly increased. The results of Micro-CT also showed that compared with the control group, the model group had experienced more alveolar bone loss and a smaller bone volume percentage. Our research results indicated that the live *L. salivarius* CCFM1332 and its postbiotics had beneficial effects on this bone damage.

This study demonstrates that both live *L. salivarius* CCFM1332 and its postbiotics exhibit significant alleviating effects in a rat periodontitis model, suggesting potential clinical implications. However, several critical aspects require careful consideration before advancing to clinical trials. First, safety assessment is paramount. Although *L. salivarius* CCFM1332 is derived from the oral cavity of healthy humans and no adverse effects were observed in rat experiments, its potential interactions with the complex human oral symbiotic microbiota need thorough evaluation. While postbiotics formulations circumvent the safety risks associated with live bacteria, rigorous standardization and quality control of their key bioactive components (such as short-chain fatty acids (SCFA), proteins, etc.) are essential. Second, optimizing the dosage form and delivery system is crucial for ensuring efficacy. To meet the requirement for local retention in periodontal pockets, live bacteria could be formulated into adhesive gels or oral lozenges; postbiotics, however, are better suited for delivery via mouthwashes or oral sprays.

It should be noted that this study has certain limitations. The rat periodontitis model cannot fully recapitulate the complex immune interaction network between the human host and microbial communities. Furthermore, the study focused solely on periodontitis induced by a single pathogen (*P. gingivalis*), whereas clinical periodontitis is typically a polymicrobial dysbiotic disease. More importantly, the underlying mechanisms require deeper elucidation. Although we have preliminarily confirmed the therapeutic efficacy of the CFS of *L. salivarius* CCFM1332 against periodontitis and observed a reduction in biofilm as an initial indicator of anti-biofilm potential, future studies should incorporate assessments of biofilm viability to elucidate the bactericidal effects. Moreover, the specific active components within the CFS remain unidentified. Future research must delve into characterizing the active components within *L. salivarius* CCFM1332 CFS. This involves quantitatively measuring the content of key constituents like SCFA and protein classes, followed by further functional validation to pinpoint the core active ingredients and their mechanisms of action. This knowledge will be vital for optimizing and validating the clinically suitable dosage forms and delivery strategies mentioned earlier for both live bacteria and postbiotics, thereby laying a solid foundation for subsequent human trials.

## 5. Conclusions

This study demonstrates the therapeutic potential of both live *L. salivarius* CCFM1332 and its postbiotics in a rat model of *P. gingivalis*-induced periodontitis. Our data reveal that intervention with these agents significantly suppressed periodontal pathogen colonization, ameliorated histopathological damage, attenuated systemic inflammatory cascades, and reduced alveolar bone loss. Notably, the live probiotics and postbiotics demonstrated comparable efficacy. These findings position *L. salivarius* CCFM1332-derived postbiotics as promising candidates for developing stable, non-living biotherapeutics against periodontitis.

## Figures and Tables

**Table 1:** The inhibition effect of different *Lactobacillus* on *P. gingivalis*.

*Lactobacillus strains*	DIZ (mm)
Positive control (0.02% CHX)	12.46 ± 0.43
Negative control	-
*L. salivarius* FHNXY73M9	-
*L. salivarius* FGSYC47M10	-
*L. salivarius* CCFM1332	20.15 ± 0.80
*L. salivarius* FXJWS6M4	-
*L. salivarius* FJSWX10-2	-
*L. plantarum* QS6-12	14.18 ± 0.63
*L. plantarum* CCFM242	11.86 ± 0.50
*L. plantarum* DL2-1	11.28 ± 0.46
*L. plantarum* CCFM10	10.82 ± 0.40

**Table 2:** Anti-biofilm activity of *Lactobacillus* against *P. gingivalis*.

*Lactobacillus*	OD_600_	Biofilm Reduction Rate (%)
Positive control (0.02% CHX)	0.18 ± 0.04	89.47
Negative control	1.71 ± 0.44	-
*L. salivarius* FHNXY73M9	0.15 ± 0.01	91.23
*L. salivarius* FGSYC47M10	0.18 ± 0.03	89.47
*L. salivarius* CCFM1332	0.12 ± 0.04	92.98
*L. salivarius* FXJWS6M4	0.25 ± 0.17	85.38
*L. salivarius* FJSWX10-2	0.17 ± 0.02	90.06
*L. plantarum* QS6-12	0.23 ± 0.02	86.48
*L. plantarum* CCFM242	0.27 ± 0.01	84.03
*L. plantarum* DL2-1	0.27 ± 0.03	83.98
*L. plantarum* CCFM10	0.23 ± 0.02	86.74

**Table 3:** Changes in body weight of rats.

Groups	Body Weight (g)					Weight Gain (g)
	7 d	14 d	21 d	28 d	35 d	
NT	253.3 ± 2.2	328.5 ± 6.0	370.4 ± 4.6	422.4 ± 1.8	457.6 ± 6.9	204.2 ± 5.2
PM	240.8 ± 9.7	304.5 ± 23.0	358.3 ± 17.0	387.9 ± 8.9	414.3 ± 5.4	173.5 ± 15.1
PC	251.9 ± 14.9	290.4 ± 16.4	374.4 ± 3.9	394.9 ± 19.9	417.6 ± 14.1	165.7 ± 23.0
PL	243.9 ± 8.2	313.3 ± 4.4	355.6 ± 4.7	391.3 ± 6.4	428.5 ± 6.4	183.3 ± 12.9
PP	254.9 ± 5.2	322.0 ± 3.6	362.3 ± 5.9	399.9 ± 7.8	423.3 ± 8.3	168.4 ± 6.5

Note: NT, no-treatment control group; PM, periodontitis model group; PC, periodontitis treated with CHX; PL, periodontitis treated with 1 × 10^9^ CFU/mL live *L. salivarius* CCFM1332 group; PP, periodontitis treated with postbiotics of *L. salivarius* CCFM1332 group.

**Table 4:** The PD and GI of rats.

Groups	PD	GI
NT	0.24 ± 0.05	0.00 ± 0.00
PM	1.24 ± 0.11 ^##^	1.50 ± 0.29 ^##^
PC	0.58 ± 0.09 **	0.50 ± 0.29 *
PL	0.85 ± 0.06 *	0.50 ± 0.29 *
PP	0.92 ± 0.09	0.60 ± 0.24 *

Note: ^##^ *p* < 0.01 vs. NT group; * *p* < 0.05 vs. PM group; ** *p* < 0.01 vs. PM group.

## Data Availability

Data are contained within the article.
